# Associative responses to visual shape stimuli in the mouse auditory cortex

**DOI:** 10.1371/journal.pone.0223242

**Published:** 2019-10-03

**Authors:** Manabu Ogi, Tatsuya Yamagishi, Hiroaki Tsukano, Nana Nishio, Ryuichi Hishida, Kuniyuki Takahashi, Arata Horii, Katsuei Shibuki

**Affiliations:** 1 Department of Neurophysiology, Brain Research Institute, Niigata University, Asahi-machi, Chuo-ku, Niigata, Japan; 2 Department of Otolaryngology, Head and Neck Surgery, Graduate School of Medical and Dental Sciences, Niigata University, Asahi-machi, Chuo-ku, Niigata, Japan; Harvard Medical School, UNITED STATES

## Abstract

Humans can recall various aspects of a characteristic sound as a whole when they see a visual shape stimulus that has been intimately associated with the sound. In subjects with audio-visual associative memory, auditory responses that code the associated sound may be induced in the auditory cortex in response to presentation of the associated visual shape stimulus. To test this possibility, mice were pre-exposed to a combination of an artificial sound mimicking a cat’s “meow” and a visual shape stimulus of concentric circles or stars for more than two weeks, since such passive exposure is known to be sufficient for inducing audio-visual associative memory in mice. After the exposure, we anesthetized the mice, and presented them with the associated visual shape stimulus. We found that associative responses in the auditory cortex were induced in response to the visual stimulus. The associative auditory responses were observed when complex sounds such as “meow” were used for formation of audio-visual associative memory, but not when a pure tone was used. These results suggest that associative auditory responses in the auditory cortex represent the characteristics of the complex sound stimulus as a whole.

## Introduction

Complex or spectrally rich sounds such as species-specific vocalization are made of elementary sound components, including pure tones, harmonic tones, amplitude-modulated sounds, frequency-modulated sounds, and noise. In order to perceive a complex sound as a whole, not as the individual sound components, the characteristics of the whole sound must be bound and coded together in the brain [1, 2], especially in the auditory cortex (AC) [3]. AC neurons show more complex selectivity for sound features compared with subcortical neurons, and the AC is increasingly understood to be an integral part of the neural networks responsible for auditory perception [3]. However, it is not well understood how a complex sound is coded as a whole in the AC. According to tonotopic maps and specific thalamic afferent projections, the AC of mice is divided into multiple areas such as the anterior auditory field (AAF), the primary auditory field (A1), and the secondary auditory filed (A2) [4–6]. Each of the auditory fields is fine-tuned to elementary sound components. Therefore, multiple components of a complex sound are expected to activate multiple auditory subfields in parallel. These individual responses in the AC must be bound together to represent the complex sound as a whole. Presumably, synchronized responses induce spike-timing dependent potentiation of neurons [7] through the well-developed horizontal connections in supragranular layers [8, 9]. Therefore, it appears that responses to sound stimuli in the AC have at least two components: individual responses elicited via parallel thalamo-cortical afferents originating in the MGB, and additional cortical activities generated by modulatory inputs from adjacent cortical sites via horizontal connections [10]. Unfortunately, it is very difficult to isolate the latter neural activities required for integration of individual responses, since auditory responses elicited via thalamo-cortical afferents dominate. If auditory responses in the AC could be induced without the involvement of thalamo-cortical afferents, neural activities required for integration may be observed in them.

The brain processes information in a distributed manner, so that multiple features of a complex sound are detected at different sites. In order to perceive a complex sound from these features, they must be integrated as a whole [11]. Humans can associate a particular sound (e.g., cat’s “meow”) with a specific shape (e.g., cat’s image) [12, 13]. When a sound is recalled based on associative memory, responses induced in the AC are likely to include not only neural activities representing each feature of the original sound but also those required for integration [11]. In our previous study, we demonstrated that audio-visual associative memory can be established between complex sound stimuli such as cat’s “meow” and visual shape stimuli after passive and synchronized exposure to the two types of stimuli [14]. Mice were pre-exposed to an audio-visual associative stimulus consisting of a visual shape stimulus and a complex sound stimulus. In these mice, diffuse responses in the AC were elicited by the visual stimulus. However, it was difficult to identify the associative auditory responses that could be included in the diffuse responses. Therefore, the differences in the responses to a test and control visual shape stimulus were calculated in each mouse, and the results were averaged in a group of mice. These results lead us to conclude that associative auditory responses were induced by presenting the test visual shape stimulus. Furthermore, we found that associative responses were detected when complex sound stimuli, but not pure tones, were used for associative memory formation. These results suggest that the associative auditory responses in the AC might represent neural activities required for integration, since integration is required only when multiple elementary components are included in the sound stimulus.

## Materials and methods

All experiments were approved by the ethics committee of animal experiments in Niigata University (approval numbers: 372–7 and SA00143), and were carried out in accordance with the approved guidelines. Male C57BL/6 mice between 5–6 weeks of age, purchased from Charles River Japan (Yokohama, Japan), were used.

### Formation of audio-visual associative memory

**Fig 1:**
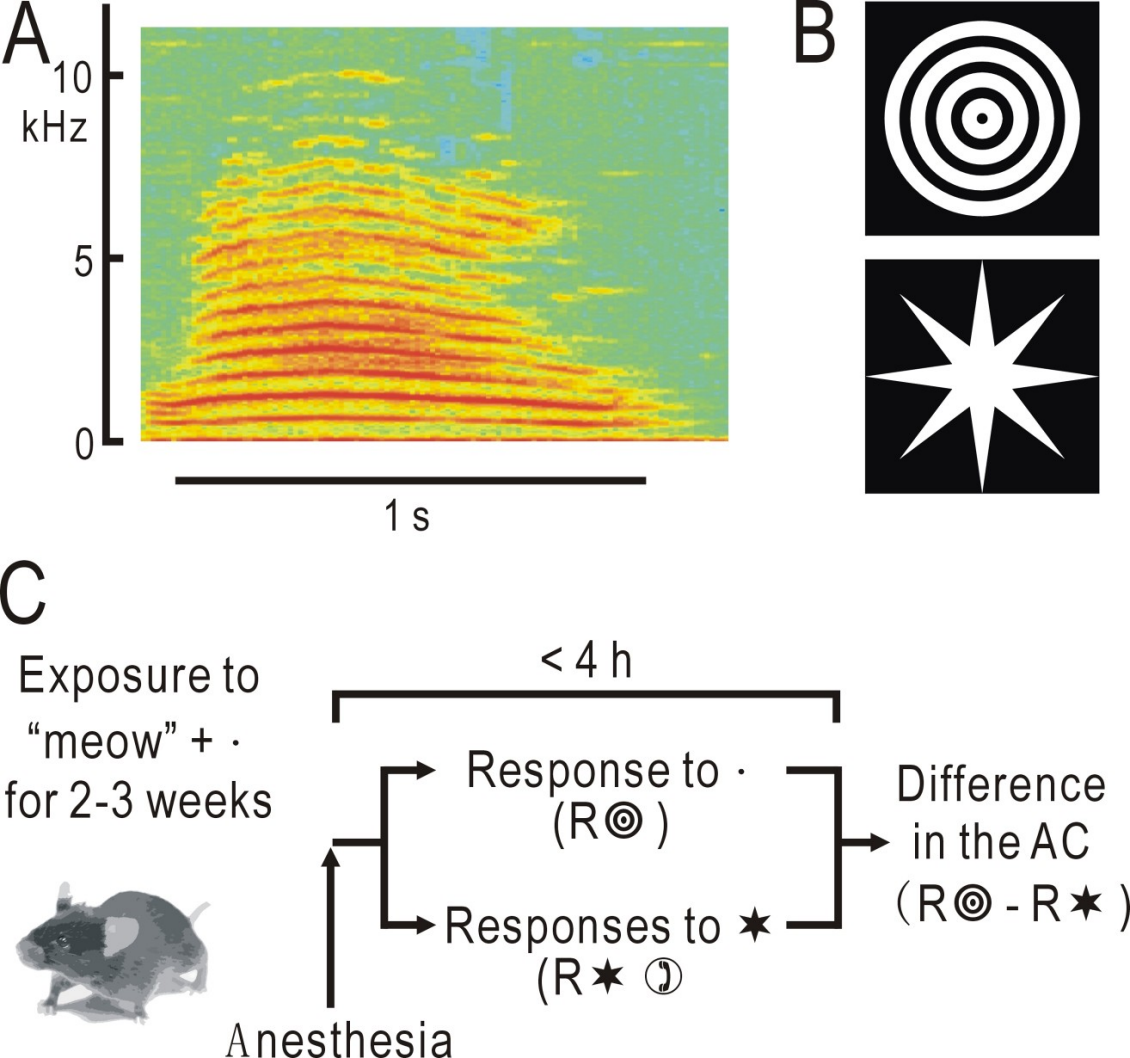
Methods for visualizing associative auditory responses. (A) Spectrogram of an artificial sound mimicking the sound of a cat’s “meow”. A part of this sound lasting 1 s (horizontal line) was used in the present study. (B) Visual shape stimuli of concentric circles with an angle of view of approximately 20°, or stars. These images were used as test or control shape stimuli. (C) Experimental schedule. Mice were pre-exposed to the test visual shape stimulus synchronized with the “meow” sound, producing an audio-visual associative memory. After the exposure, mice were anesthetized and responses to the test visual stimulus and the other control shape stimulus were recorded in the AC. The difference between the responses was obtained in each mouse and averaged in a group of mice that received the same experimental procedure (n = 12–18 for each group).

To establish an audio-visual associative memory, mice were passively exposed to synchronized audio-visual stimuli [14]. Mice between 5–6 weeks of age were bred for 2–3 weeks in a mouse cage composed of transparent acrylic plates placed in a soundproof box. The cage was kept at a temperature of 22–24° C and humidity of 50–70%. Water and dry pellets were available ad libitum. Four visual display monitors of 8 inches (LCD8000, Century, Tokyo, Japan) were placed around the mouse cage, and a speaker (Companion 2, Bose, Framingham, USA) was placed on top of the cage. To establish an audio-visual association, the audio- and visual stimuli were presented simultaneously five times, for a duration of 1 s and at 2 s intervals. These audio-visual stimuli were repeated at 30 s intervals throughout the breeding in the dark box. The complex sound stimulus, an artificial sound mimicking a cat’s “meow” (Fig 1A), was obtained from a free online source (http://taira-komori.jpn.org/index.html). We also used four other types of sound stimuli: a pure tone at 5 kHz, a frequency-modulated (FM) tone with the frequency linearly reduced by 50% from 5 kHz to 2.5 kHz per second, a harmonic tone at 3, 4, 5, 6 and 7 kHz, and a FM harmonic tone with the frequency of each component reduced by 50% per second. In each case, the rise/fall time of 10 ms was set and the maximum sound pressure was adjusted to be 70 dB SPL at the center of the cage. For the visual stimuli, we used concentric circles or stars with an angle of view of approximately 20° (Fig 1B). To synchronize the timing of the sound and visual stimuli, we produced an audio-visual movie file using the animation function of the PowerPoint (Microsoft), the file lasted 10 s during which sound and visual shape presentation were synchronized. The presentation of the movie file was repeated at 30 s intervals during breeding in the mouse cage.

### Transcranial flavoprotein fluorescence imaging

Transcranial imaging of endogenous green fluorescence derived from mitochondrial flavoproteins was used for recording cortical activities in the AC; this imaging technique has been proven appropriate for investigating cortical plasticity in the AC [15, 16] and other brain areas in mice [17–19]. Surgical procedures were conducted under sterile conditions, as described previously [20, 21]. We anesthetized mice with urethane (1.65 g/kg, i.p.). Rectal temperature was monitored and maintained at 37.5°C using a silicon rubber heater throughout recordings. The skin covering the target area was incised after subcutaneous injection of bupivacaine (AstraZeneca, Osaka, Japan). The temporal muscle over the right AC was removed. A piece of metal was attached to the skull using acrylic dental resin (Super Bond; Sun Medical, Shiga, Japan), and the head was fixed in place by screwing the metal piece onto a manipulator. The exposed surface of the intact skull was covered with liquid paraffin to prevent drying and to keep the skull transparent. The operation was finished within 30 min. Recordings were performed between 90 min and 4 h after introducing anesthesia, since the status of anesthetized mice was usually stable during this time. At the end of the imaging experiments, the mice were euthanized with an overdose of pentobarbital (300 mg/kg, i.p.).

A camera (ORCA-ER, Hamamatsu Photonics, Hamamatsu, Japan) was attached to a binocular epifluorescence microscope (M651 combined with MZ FL II, Leica Microsystems, Wetzlar, Germany) with a 75 W xenon light source. Green fluorescence images (λ: 500–550 nm) excited by blue light (λ: 450–490 nm) were recorded in cortical areas including the right AC, while the mice were exposed to visual or sound stimuli in trials repeated at 30 s intervals. Images (128 × 168 pixels after binning) were averaged over 20–30 trials using an image processor (Aquacosmos system, Hamamatsu Photonics). Spatial moving averaging in 5 × 5 pixel areas and temporal moving averaging in three consecutive frames were also used to improve the image quality. The obtained images were normalized, pixel by pixel, with respect to a reference image (F_0_), which was formed by averaging three images obtained immediately before stimulus presentation. The one second sections of auditory or visual stimulus used for establishing the audio-visual associative memory were also used for the imaging experiments. The normalized amplitudes of changes in flavoprotein fluorescence (ΔF/F_0_) were estimated using a circular region of interest (ROI; diameter: 30 pixels or 0.6 mm). The center of the ROI was placed at the AAF, where characteristic patchy responses were recorded in response to the sound mimicking “meow”. The images obtained from different mice that received the same experimental procedure were averaged using a MATLAB program developed by us. The center of the ROI in AAF of each image was used as the reference point for superimposing and averaging the images between different mice, as the functional identification of AAF was easily achieved in each mouse using the characteristic localized patchy responses. When visual stimuli were used, the small responses in the AC were strongly affected by baseline fluorescence changes as a result of photobleaching and visual responses in the ventral part of the visual cortex. To cancel out this interference, the differential AC responses to a pair of visual stimuli (concentric circles vs. stars) were estimated in each mouse, and the results were averaged within a group of mice that received the same experimental procedure (Fig 1C).

### Statistical analysis

Data were analyzed using a one-way ANOVA in Easy R, free software for statistical analysis [22]. In post hoc analysis, data obtained in different groups of mice were analyzed using a t-test. P values were corrected for multiple comparisons using the Bonferroni method. Only P values less than 0.05 are shown. Values in the figures represent the mean and SEM in groups of mice, unless otherwise specified.

## Results

**Fig 2:**
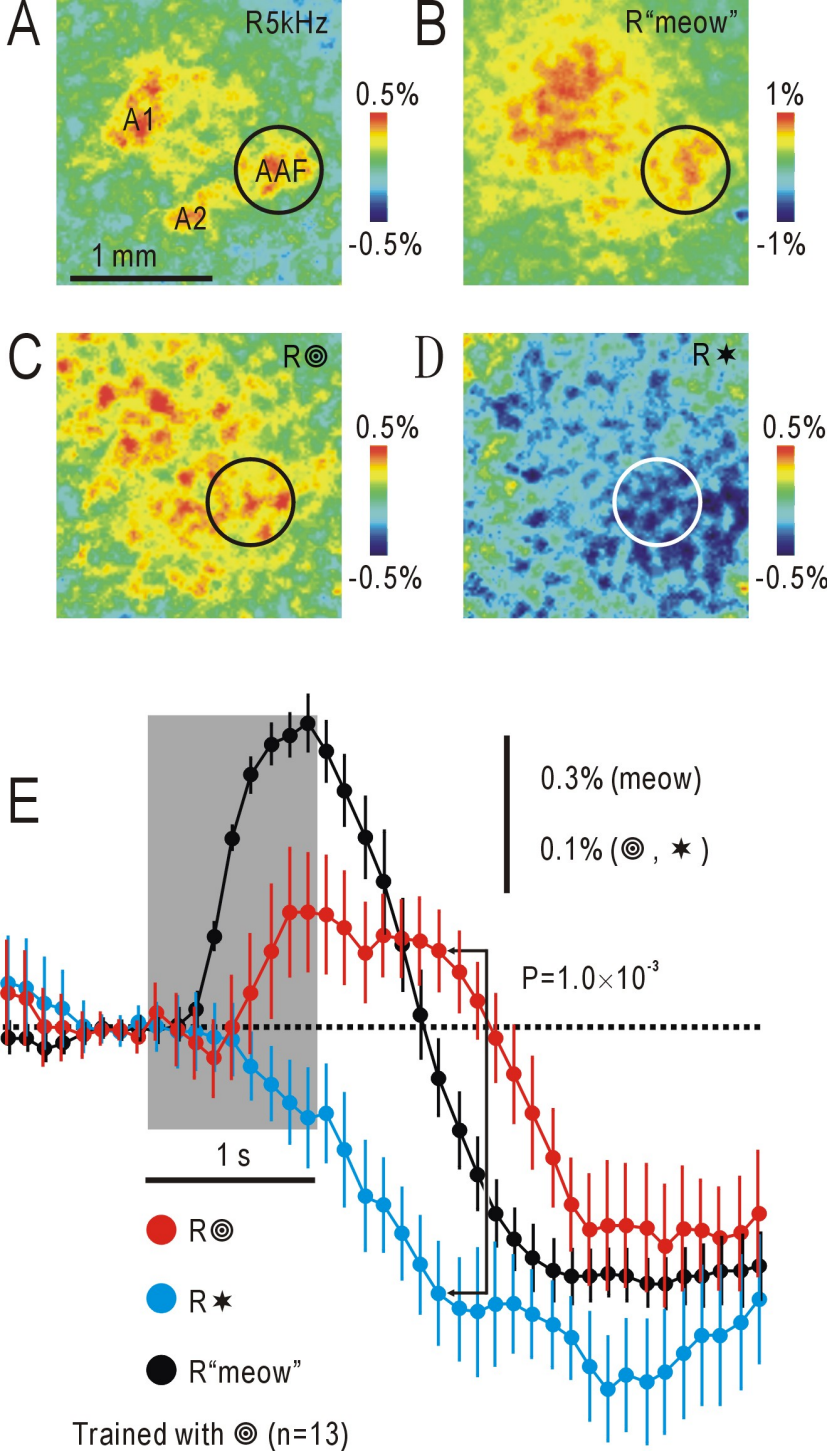
Responses to auditory or visual shape stimuli in the AC. (A) Image of responses to a pure tone at 5 kHz in the right AC (R5kHz). A1, AAF, and A2 were functionally identified. Circle shows patchy responses in AAF. Upper right corner in the panel corresponds to medial and anterior areas of the cortex. The pseudocolor scale shows ΔF/F_0_. (B) Responses to a cat’s “meow” sound (R“meow”). The circular ROI shows the location of the characteristic patchy response in AAF. Data shown in (A) and (B) were obtained from the same mouse, and recorded 0.5 and 1 s after stimulus onset, respectively. (C) Responses to concentric circles (test shape stimulus) (R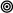). Diffuse increases in ΔF/F_0_ were observed in the AC. (D) Responses to stars (control shape stimulus) (R✶). Diffuse decreases in ΔF/F_0_ were observed in the AC. Data shown in (C) and (D) were obtained from the same mouse, and recorded 1.8 s after stimulus onset. (E) Time courses of ΔF/F_0_ within the circular ROI centered on AAF in 13 mice. Dots with lines show means and SEMs. Vertical scale bar: 0.3% for ΔF/F_0_ in response to the “meow” sound, 0.1% in response to visual shape stimuli. Shaded square shows the timing of stimulus presentation.

Mice were pre-exposed to an audio-visual associative stimulus consisting of concentric circles and a “meow” sound for at least two weeks. After anesthesia, control responses to a pure tone at 5 kHz were recorded in these mice. Responses to the 5 kHz sound (Fig 2A) were maximal at approximately 0.5 s after stimulus onset, because the responses were attenuated during stimulation. Based on typical responses in the AC [5, 6], auditory areas including A1, AAF, and A2 were functionally identified (Fig 2A). When presented with the “meow” sound, responses were observed in a wider area of the AC, with a patchy response in AAF (circles in Fig 2A and 2B). The responses to the “meow” sound (Fig 2B) were maximal at approximately 1 s after stimulus onset, because the responses were less easily attenuated and the spectrogram of the “meow” sound (Fig 1A) has a complex time course. We set a circular ROI centered on the responses in AAF, and the time course of ΔF/F_0_ changes was investigated (Fig 2E). Transient responses were observed during presentation of the “meow” sound. On the other hand, when mice were stimulated by visual shape stimuli, such as the test shape stimulus of concentric circles or a control stimulus of stars, diffuse changes in ΔF/F_0_ were found in the AC (Fig 2C and 2D). When the time courses of ΔF/F_0_ were compared between the two conditions (Fig 2E), differences in ΔF/F_0_ were found between the response to circles and that to stars. The amplitude of the difference was largest approximately 1.8 s after stimulus onset (Fig 2E). We recorded 33 images after stimulus onset. Although there was no solid reason to choose a particular time point for measuring amplitudes of associative responses, we must fix the timing of measurement to avoid overestimation resulted from baseline fluctuation. Therefore, we arbitrarily selected the time point at 1.8 s after stimulus onset in statistical analysis, and P values were corrected for multiple comparisons between 33 image pairs recorded after stimulus onset using the Bonferroni method.

**Fig 3:**
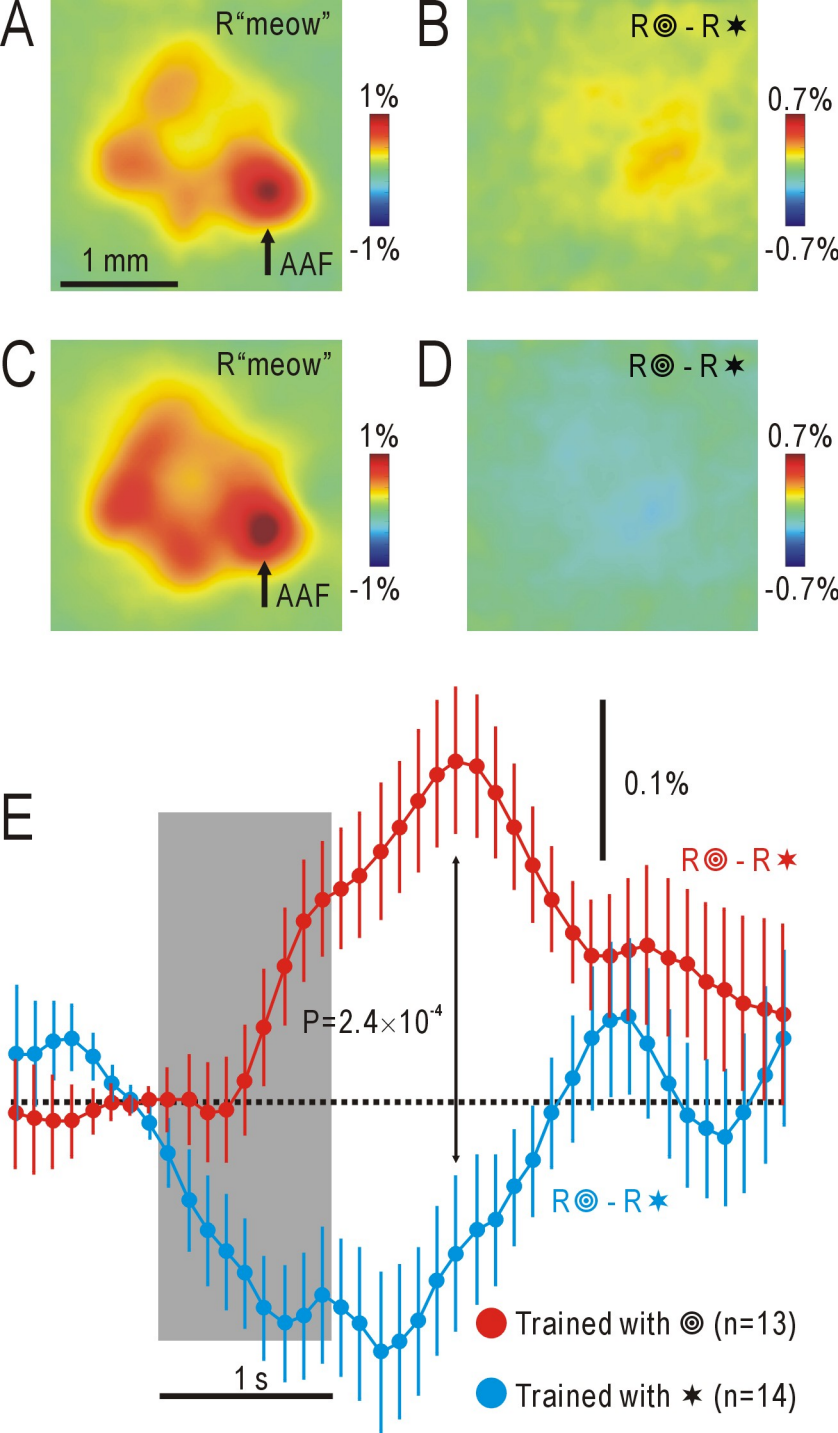
Averaged responses to auditory or visual shape stimuli in the AC. (A) Averaged responses in the AC to the “meow” stimulus in 13 mice pre-exposed to a combination of the “meow” sound and concentric circles (R“meow”). Arrow shows the position of AAF. The pseudocolor scale shows ΔF/F_0_. (B) Associative auditory responses calculated as the differential responses to concentric circles (test) and stars (control) in the same 13 mice shown in (A) (R
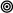
—R✶). Note the responses with positive amplitudes. (C) Averaged responses in the AC to the “meow” stimulus in 14 mice pre-exposed to a combination of the “meow” sound and stars (R“meow”). (D) Associative auditory responses calculated as the differential responses to concentric circles (control) and stars (test) in the same 14 mice shown in (C) (R
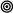
—R✶). Note the responses with negative amplitudes. Data shown in (A) and (C) were recorded 1 s after stimulus onset, and those in (B) and (D) were recorded 1.8 s after stimulus onset. (E) Time courses of ΔF/F_0_ within the circular ROI centered on AAF in (B) and (D). Dots with lines show means and SEMs.

When concentric circles were presented, diffuse increases in ΔF/F_0_ were observed throughout the AC and a ventral part of the visual cortex (Fig 2C). It was difficult to identify the precise distribution of the associative auditory responses elicited by the stimuli. Therefore, in each mouse, the differences in the responses to a test and control visual shape stimulus were calculated, and the results were averaged in a group of mice that received the same experimental procedure (Fig 1C). The averaged response to the “meow” sound (Fig 3A) was essentially similar to the responses observed in each mouse (for example, Fig 2B), and details of the activated auditory areas were more clearly identified in Fig 3A, demonstrating the effectiveness of the averaging procedure. The associative auditory responses to visual shape stimuli were also clearly observed in the averaged images, in which localized responses were observed in AAF and surrounding areas of the AC (Fig 3B and 3E).

The differential responses in Fig 3B might be a result of a difference in innate sensitivity of the AC to concentric circles and stars. To exclude this possibility, the differential responses were investigated in another group of mice that had been pre-exposed to audio-visual associative stimuli of the “meow” sound and stars. In these mice, auditory responses to the “meow” sound were similar to those recorded in mice pre-exposed to associative audio-visual stimuli of concentric circles and the “meow” sound (Fig 3C). However, concentric circles and stars elicited differential responses with negative amplitudes in these mice (Fig 3D and 3E). These results indicate that the previous experience has a significant effect on the differential AC responses, although they do not exclude the presence of any innate difference between AC responses to circles and stars. These results strongly suggest that the differential AC responses shown in Fig 3B, 3D and 3E were a result of the auditory response to the “meow” stimulus recalled by presentation of the visual shape stimuli that had been associated with the “meow” sound.

**Fig 4:**
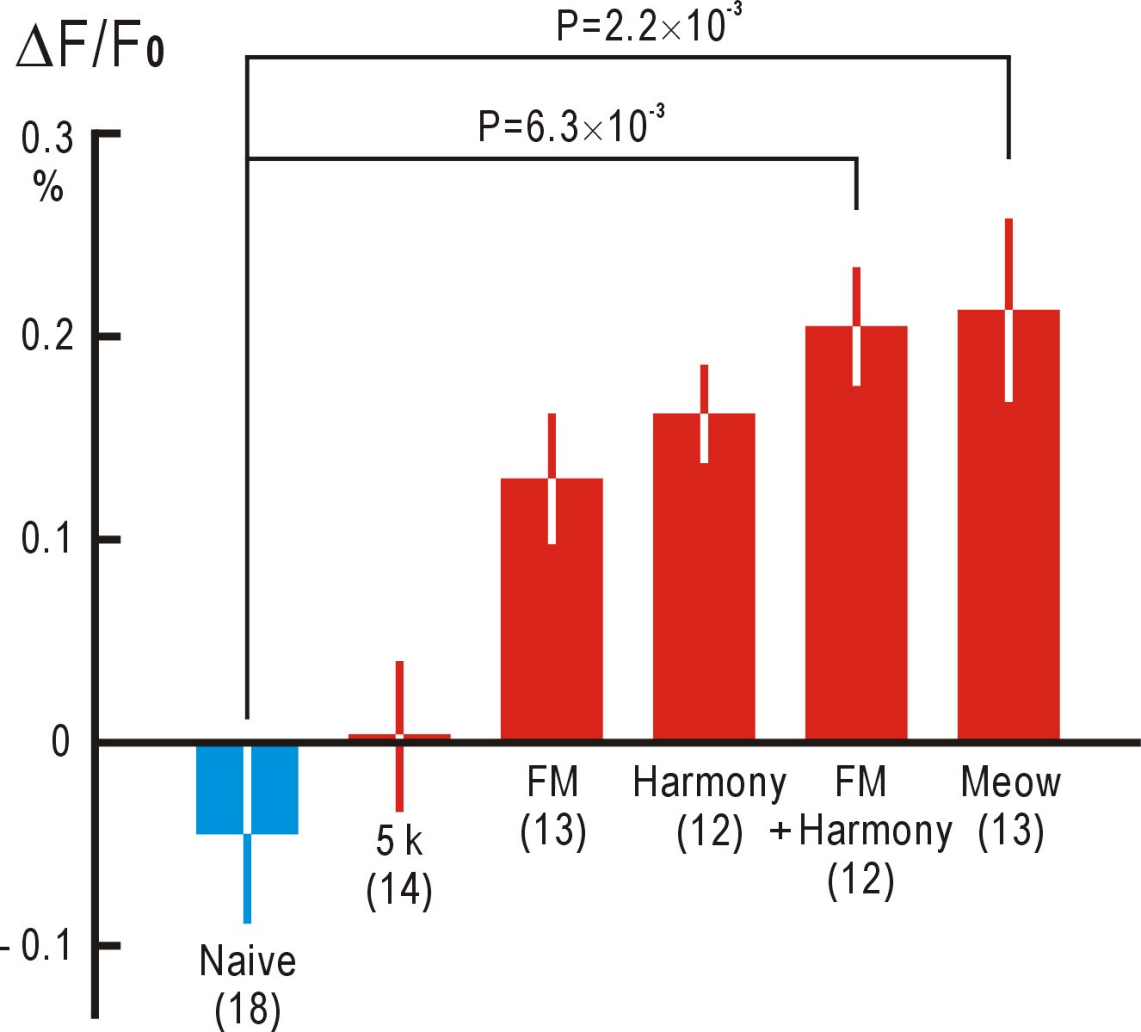
Associative auditory responses in the AC to visual stimuli. Amplitudes of the associative responses to a 5 kHz pure tone, FM tone, harmonic tone, FM harmonic tone, and a “meow” sound (red columns). Columns with lines show means and SEMs. Concentric circles and stars were used as visual shape stimuli. Amplitudes of control responses with no pre-exposure are shown by a blue column.

We used a complex and characteristic artificial sound mimicking “meow” in this experiment. We further analyzed how the complexity of sound stimuli might affect associative memory responses in the AC. When a pure tone at 5 kHz was used instead of the “meow” stimulus, no differential AC response (difference between AC responses to circles and stars) was recorded (Fig 4). However, when a FM sound that linearly changed frequency from 5 to 2.5 kHz in one second was used, differential AC responses were observed, although the amplitudes were not significantly larger than those of naive mice that had not been exposed to any audio-visual associative stimuli (Fig 4). When a harmonic sound at 3, 4, 5, 6, and 7 kHz was used, slightly larger but not significant differential AC responses were recorded. A harmonic FM sound, in which the frequency of each component linearly decreased by 50% in one second, produced even larger and significant differential AC responses. The differential AC responses to “meow” were larger than those to the harmonic FM sound. Statistical significance of P = 7.6×10^−5^ was found in these data (one-way ANOVA). In post-hoc analysis, statistical significance of the differences between the amplitudes of each exposed group was also evaluated using a t-test with the Bonferroni correction for multiple comparisons between the exposed groups (red columns in Fig 4) and the naive control group (blue column). These results strongly suggest that the complexity characterizing sound stimuli is required to produce associative auditory responses.

## Discussion

### Imaging associative auditory responses

In the present study, associative auditory responses to presentation of visual shape stimuli were recorded using mitochondrial flavin protein fluorescence reflecting activity-dependent oxygen metabolism in neurons [23, 24]. The amplitudes of the associative responses (approximately 0.2% as ΔF/F_0_) were much smaller than those reported previously (approximately 1%) using this technique [5, 6, 15, 16, 20]. Since these associative responses reached to a peak 1.5–2 s after stimulus onset, they were obscured by photobleaching and activity-dependent hemodynamic responses that appeared approximately 1 s after stimulus onset [25]. Furthermore, since we investigated visually-induced associative responses, weak responses in the AC were interfered by visual responses in the ventral part of the visual cortex. To overcome these difficulties, we used a pair of visual shape stimuli, associated and non-associated, and the difference between the responses to the two stimuli were recorded in each mouse (Fig 1C). Furthermore, the results obtained in each mouse were averaged within the group of mice that received the same experimental procedure. For averaging between different mice, it was necessary to align the cortical recording position in each mouse. We used the center of the localized patchy responses to the “meow” stimulus in AAF as the standard point of alignment, since these responses were easily identifiable in each mouse (for example, Fig 2B) Using this methodology, we succeeded in recording changes in visually-induced associative auditory responses that were as small as 0.2% in amplitude. However, rotational variation in the AC of each mouse was not corrected, so responses in AAF could be exaggerated by the averaging procedure. However, details of the average control responses to the “meow” stimulus (Fig 3A and 3C) were more clearly identified when compared with those of the original responses (Fig 2B). The recent development of Ca^2+^-sensitive fluorescent proteins [26–28] is useful for visualizing slight changes in mouse cortical activities that are difficult to visualize with flavoprotein fluorescence imaging (for example, [29]). However, it is difficult to exclude the possibility that exogenous Ca^2+^-sensitive fluorescent proteins might interfere with experience/Ca^2+^-dependent changes in cortical functions. The present results indicate that fluorescence imaging using endogenous flavoproteins may still have some merits for investigating experience-dependent changes in higher cortical functions when combined with averaging of the obtained data in a group of mice.

### Presence of associative auditory responses

Associative learning is known to be induced between multisensory inputs [14, 30]. Such learning can be attributed to the functions of NMDA receptor-dependent long-term potentiation (LTP) [31–33] or spike-timing dependent LTP [7] when two types of stimuli induce synchronized neural activities. NMDA receptor-dependent LTP has also been observed in the AC [34, 35]. Previously, we have demonstrated that mice can perform a match to sample test using two kinds of visual shape stimuli [14]. During training, each sample shape was repeatedly presented together with a sound stimulus specific to each shape. At test, presentation of sample shapes could be replaced by presentation of the associated sound stimuli with almost similar test performance [14]. These results suggest that mice can recall sample visual shapes following presentation of the associated sound stimuli. Therefore, we predicted that mice would recall associated sounds following presentation of the visual shape stimuli. The present results confirmed this prediction; associative auditory responses were observed in the AC following presentation of the visual shape stimuli. In our previous study [14], an audio-visual associative memory has been established by synchronized exposure to visual shape and auditory stimuli for 48 h. We used an exposure time of 2–3 weeks to produce an associative memory that was detectable in anesthetized mice. The present results indicate that the AC can be activated without the involvement of thalamic inputs from the MGB. These results suggest that higher auditory responses can be induced without being disturbed by the direct responses to sound stimuli, and analysis of the higher responses may be important for elucidating higher functions in the AC.

### Cortical representation of complex sounds

There are various arguments regarding how complex sensory stimuli are coded, especially in the visual system. One possibility is that the firing of specific neurons responding to a complex sound stimulus represents the sound in the brain, similar to “grandmother cells” in the visual system [36, 37]. The number of such neurons decrease as the complexity of stimuli is increased. An alternative hypothesis is that synchronized firing of neurons coding different aspects of the complex stimulus represents the stimulus as a whole [38, 39]. According to the latter hypothesis, the number of neurons coding the stimulus must increase as the complexity of stimulus is increased. At present, little is known about how complex sound stimuli are coded in the AC. In the present study, we found that a complex sound stimulus was required to induce associative auditory responses, and that associative responses to more complex stimuli were larger. These results are compatible with the idea that the synchronized firing of many neurons represents complex stimuli [38, 39]. Possibly, the associative auditory responses represent cortical activities in the supragranular layers, since cortical responses in the deeper layers are difficult to record optically [40]. In addition to thalamic inputs originating in the MGB, supragranular areas are driven by cortico-cortical projections [41] and well-developed horizontal connections in the AC [8–10]; it is likely that associative auditory responses are produced by these inputs. High complexity of the sound stimulus was required to induce associative auditory responses. Our results raise a hypothesis that associative auditory responses might include neural activities required for integrating various aspects of the original complex sound as a whole.

## Supporting information

S1 Data Data set for Fig 2E. ΔF/F_0_ values are shown in percentages.
(XLSX)

S2 Data Data set for Fig 3E. ΔF/F_0_ values are shown in percentages.
(XLSX)

S3 Data Data set for Fig 4. ΔF/F_0_ values are shown in percentages.
(XLSX)
